# ICF‐Based Assessment of Functioning Problems in Parkinson’s Disease: Findings From a Cross‐Sectional Survey in Southern Ghana

**DOI:** 10.1155/padi/4054078

**Published:** 2026-05-29

**Authors:** Mary Wetani Agoriwo, Erika Franzén, Marianne Unger, Conran Joseph

**Affiliations:** ^1^ Division of Physiotherapy, Department of Health and Rehabilitation Sciences, Faculty of Medicine and Health Sciences, Stellenbosch University, Cape Town, South Africa, sun.ac.za; ^2^ Department of Physiotherapy and Rehabilitation Sciences, School of Allied Health Sciences, University of Health and Allied Sciences, Ho, Ghana, uhas.edu.gh; ^3^ Division of Physiotherapy, Department of Neurobiology Care Sciences and Society, Karolinska Institutet, Stockholm, Sweden, ki.se; ^4^ Medical Unit Allied Health Professionals, Theme Women’s Health and Allied Health Professionals, Karolinska University Hospital, Stockholm, Sweden, karolinska.se

**Keywords:** activity limitation, barriers, facilitators, functioning problems, ICF, impairments, Parkinson’s disease, participation restrictions

## Abstract

**Background:**

Most assessment tools may not be comprehensive enough to measure all relevant problems associated with Parkinson’s disease (PD) and may focus only on activity. While specific performance‐based outcome assessments are crucial for intervention design, it is important to establish the epidemiology of functioning problems, to understand the broad range of disability associated with a disease.

**Objectives:**

This study aimed to describe the prevalence and nature of functional problems and the contextual factors experienced by persons with PD (PwPD) using the International Classification of Functioning, Disability and Health (ICF) framework. The distribution of substantial functioning problems across selected demographic and clinical variables was also assessed.

**Methods:**

A cross‐sectional survey was conducted among PwPD attending one primary and two tertiary hospitals in southern Ghana. The ICF Checklist (Clinician Form) was adapted to record impairments, activity limitations, participation restrictions, and environmental facilitators and barriers. Descriptive and inferential analyses were performed, with significance set at *p* < 0.001 for multiple comparisons using the Hochberg correction.

**Results:**

The study included 75 PwPD (61.3% male) with a mean (SD) age of 66.8 (9.6) years. The most frequently reported issues were impairments in body functions and participation restrictions, with the top 10 problems reported by 68%–91% and 67%–88% of participants, respectively. Participants reported more facilitators, while challenges with general social support were the most frequently reported barrier among 60% of participants. Women and individuals with advanced PD were significantly more likely (*p* < 0.001) to report substantial functioning problems.

**Conclusion:**

This study highlights critical functioning problems faced by PwPD in a resource‐limited setting and underscores the value of using the ICF framework in rehabilitation assessment and planning. The findings may inform gender‐ and disease‐stage‐sensitive interventions for PwPD across diverse healthcare contexts.

## 1. Introduction

Parkinson’s disease (PD) is recorded as the second most common neurodegenerative condition worldwide [1, 2] and a rapidly growing neurological condition causing death and disability [3]. PD presents with a wide spectrum of motor (bradykinesia, resting tremors, rigidity, poor balance, and so on) and nonmotor (cognitive impairments, autonomic system, behavioral disorders, and so on) symptoms with a severe effect on patients’ physical activity levels, cognitive functioning, and quality of life [4, 5]. This results in difficulties with basic human functioning in activities of daily living and participation such as walking, grooming, swallowing, working, attending social events, and difficulty maintaining relationships with family and friends, among others [6].

Due to the complexity of PD, effective assessment of these functioning problems is critical for both medical and rehabilitation interventions. Previous studies have recommended the use of several outcome measures such as the Berg Balance Scale, Activities‐specific Balance Confidence Scale, Functional Gait Assessment Scale, 10‐m Walk Test (10MWT), and the 6‐Minute Walk Test (6MWT) in assessing impairments and levels of activity and function in persons with PD (PwPD) [7]. However, most of these assessment tools may not be comprehensive enough to measure all the relevant problems associated with PD [8], particularly in contexts where rehabilitation services and access pathways are inconsistent. Many commonly used outcome measures emphasize impairments and discrete performance tasks, with limited attention to activity, participation, and the contextual factors that shape everyday functioning. In low‐ and middle‐income settings, where environmental, social, and health system factors play a critical role in determining rehabilitation experiences and outcomes, a framework that extends beyond the impairment‐level assessment is required. The International Classification of Functioning, Disability and Health (ICF) offers such a framework, enabling a systematic and context‐sensitive description of functioning and disability to inform intervention planning.

To address these limitations, the ICF framework provides a comprehensive guide for assessing disability across numerous conditions [9]. Unlike impairment‐focused outcome measures, the ICF adopts a biopsychosocial approach that integrates body function and structure impairments, activity limitations, participation restrictions, and the contextual factors (personal and environmental) that shape everyday functioning. This is particularly relevant in settings where access to rehabilitation services, assistive devices, and social support is variable, and where environmental and health system factors strongly influence lived experience and outcomes. The ICF also offers a common language that stakeholders, including clinicians, policymakers, and persons with disabilities, can share, facilitating communication, service planning, and comparison across settings [10]. This underscores the relevance of the ICF in analyzing the functioning problems of PwPD across body function and structure impairments, activity limitations, and participation restrictions, while also accounting for the contextual factors (personal and environmental) that shape individual functioning [9]. In Europe, the ICF has been successfully applied to describe the full spectrum and severity of functioning problems across multiple health conditions, including PD [11]. In Africa, however, its application to PD remains unexplored; while the ICF has been used to assess functioning poststroke in South Africa [12], no study to date has applied the framework to describe the functioning problems of PwPD on the continent. PD prevalence is projected to increase by approximately 292% in sub‐Saharan Africa by 2050 [13], and Ghana reports PD as one of the most common hypokinetic neurological conditions [14, 15]. Therefore, there is an urgent need for a framework capable of capturing functioning problems beyond impairment alone. Applying the ICF in this context may identify priority functioning challenges relevant to people living with PD in Ghana and similar settings, thereby informing more targeted and contextually appropriate rehabilitation interventions within the constraints of a publicly funded healthcare system. The use of the ICF further enables meaningful comparison with international data where the framework is already operationalized in clinical practice.

Therefore, this study aimed to describe the (a) prevalence of functioning problems (impairments, activity limitations, and participation restrictions) and contextual factors among PwPD using the ICF framework, (b) substantial functioning problems reported among PwPD, and (c) determine differences in the substantial functioning problems stratified by gender, age, disease stage and duration, and use/nonuse of rehabilitation.

## 2. Methods

### 2.1. Study Design and Study Site Description

The study used a cross‐sectional design. Three study sites that run specialized neurology and PD‐specific clinics were purposefully selected from the southern sector of Ghana and included two teaching hospitals located in urban settings (teaching hospitals 1 and 2 [TH1 and TH2]), and one primary healthcare facility in a rural setting (primary facility [PF]). TH1 is a 2000‐bed capacity hospital located within the capital city of Ghana. It is the largest teaching and referral hospital [16, 17]. TH2 is a 240‐bed capacity hospital situated in the regional capital. These teaching hospitals run neurology clinics, which manage all types of adult neurological conditions including PD. The PF is the only primary healthcare facility in Ghana that provides PD‐specific services. It is a 70‐bed capacity hospital that also provides all forms of primary care services [18]. The Consensus‐Based Checklist for Reporting of Survey Studies (CROSS) was used as a guide to ensure the study was sufficiently reliable, reproducible, and transparent [19].

### 2.2. Study Population and Sampling

PwPD receiving care at the selected healthcare facilities were the target population for this study. Patients with a confirmed diagnosis of idiopathic PD at any disease stage based on the UK PD Society Brain Bank criteria for diagnosing PD [20] or the International PD and Movement Disorders Society (MDS) clinical diagnostic criteria for PD [4] were included. Adults with PD who scored more than 24 on the Mini‐Mental State Examination (MMSE) were considered cognitively stable and included in the study [21]. Persons with atypical and secondary Parkinsonism were excluded. A quota sampling was first used to allocate specific sample sizes to each study site based on the weekly attendance of PwPD at the clinics, 50% of the study sample from TH1, 12.3% from TH2, and 24.7% from PF. A convenient sampling method was then used to recruit PwPD. On average, 15, 5, and 10 PwPD attended the TH1, TH2, and PF clinics, respectively, per month. Data collection was planned to take place within three months at each study site, resulting in 90 (TH1 = 45; TH2 = 15; PF = 30) PwPD as the total population. Per the clinic attendance register and the anticipated duration for data collection at each site, the study sample was estimated at 74 using Yamane’s formula: *n* = *N*/(1 + Ne^2^), where *N* = population (90) and *e* = 0.05, at 95% confidence interval, *n* =  *N*/(1 + *N* *e* ^2^) = 90/1 + 90 (0.05^2^) = 90/(1 + 0.225) = 73.4 [22].

The overall data collection occurred from March to July 2024.

### 2.3. Instrument for Data Collection

The ICF Checklist Clinician Form and the “Brief Health Information” section of the Checklist [23] were adapted to design a questionnaire that collated information on participants’ demographics, functioning problems (impairments, activity limitation, and participation restriction), contextual factors (personal and environmental), and utilization of rehabilitation services. The ICF Checklist was used to structure data collection, consistent with earlier studies [6, 12]. However, the authors recognize that WHO has since published the ICF Practical Manual [24], which provides updated guidance. The ICF Checklist is not disease‐specific and not exhaustive. Therefore, unrelated entities to PD, such as learning to read (d140), write (d145), or calculate (d150), were removed, and additional relevant entities were included (Supporting Information 1). The additional entities drawn from the main ICF document were added based on functioning problems identified from an earlier scoping review [25], patient and public involvement (PPI), motor and nonmotor symptoms, and motor complications from the MDS Unified PD Rating Scale (MDS‐UPDRS). This resulted in a total of 122 entities (33 for body function component, 6 for body structure component, 44 for activities and participation components, and 39 for environmental factors).

The ICF Checklist recommends assessment and includes data from the past 1 month (30 days) [23]. Participants are rated on a scale of 0–4 challenge (0 = no challenge; 1 = mild challenge present in < 25% of the time with a tolerable intensity and occurs rarely; 2 = moderate challenge in < 50% of the time with an intensity that interferes with daily life and occurs occasionally; 3 = severe challenge present in > 50% of the time with an intensity that partially disrupts daily life and occurs frequently; and 4 = complete challenge present in > 95% of the time with intensity that totally disrupts daily life and occurs every day). The challenges may include impairments in body function and structure (e.g., rigidity, tremors, and joint stiffness), difficulties with activities and participation (e.g., challenges with walking, dressing, or engaging in social activities), and environmental barriers (e.g., limited family support, societal attitudes, and norms). Facilitators are categorized as none (0), mild (+1), moderate (+2), substantial (+3), and complete (+4). There are additional scorings of 8 for “Not specified,” where there are insufficient details to score the severity of the problem, and 9 for “Not applicable,” where it is inappropriate to apply a particular entity (e.g., b650 Mensuration functions for women in menopausal age). Activity limitation (capacity) refers to the limitation in performing specific tasks, while participation restriction (performance) refers to the difficulty in performing specific tasks [23].

### 2.4. Piloting of Researcher‐Developed Questionnaire

The adapted questionnaire was piloted among eight PwPD (four males) who were not included in the study but were receiving care at a local neurology clinic and physiotherapy department to ensure the face and content validity of the questions. See Supporting Information 2 for demographic details of pilot participants. Study participants were similar to pilot participants in terms of gender, age, disease stage and duration, and level of education. No changes were made to the questionnaire after the pilot, but the researcher gained better insights into how to interpret the questions and understand the participants’ responses. The entire questionnaire took about 30–45 min to complete.

### 2.5. Participant Recruitment and Procedure for Data Collection

Health professionals (neurologists, physician specialists, and nurses) who run the clinics discussed the study with patients and asked for their interest in participating. The study was also advertised through posters at the neurology/PD clinics of the selected facilities to raise awareness of the study. Those who expressed interest in participating were referred to the researcher for recruitment. Patients were given the information sheet, and their telephone/mobile phone numbers were taken for follow‐up calls to address any questions about the study and discuss a favorable time and day for data collection. The venue for data collection was based on participants’ preference, either at their home or at the neurology/PD clinic. On the day of scheduled data collection, informed consent was obtained before administering the questionnaires and after the study’s aims and objectives were explained. All information on body structure and function, activity and participation, and facilitators or barriers was obtained from the participants.

### 2.6. Data Processing and Analysis for Quantitative Study

Data were anonymized by assigning specific reference numbers to participants and were saved on a password‐protected computer to ensure confidentiality. To minimize data entry errors, a structured form designed in Excel and approved by the research team was used for the data entry immediately after each survey. The data sheet was constantly checked for appropriateness, and any missing data were filled in by cross‐checking with the survey forms. The Statistical Package for Social Sciences (SPSS), Version 29.0, was used for data analysis. Descriptive statistical analysis was performed on all data on demographic and ICF scores and presented as frequencies, percentages, and means with standard deviations. Findings were illustrated with tables and charts. To address the confusion with the activity and participation entities, the WHO recommendation to treat each entity as a single, fully overlapping list was adopted [23, 24]. Hence, each entity was interpreted as an activity (individual functioning) and participation (functioning within society). To avoid redundancy, the activity was measured with the capacity qualifier and participation measured with the performance qualifier. Data normality for age and disease duration was tested with the Shapiro–Wilks test. The differences among the participants from the three study sites were tested with ANOVA, F, for age (Shapiro–Wilks test = 0.99; *p* = 0.77), Fisher’s exact test, for gender and disease stage, and Kruskal–Wallis test, H, for disease duration (Shapiro–Wilks test = 0,90; *p* < 0.001).

To assess the extent of the functioning challenges and differences among study participants, the ICF scores were dichotomized as substantial (2, 3, and 4 scores) and nonsubstantial (0, 1, and 9 scores) problems. These categorical classifications were compared within different groups of selected variables: gender (male and female), age (≤ 60 and > 60), disease stage (advanced, Hoehn and Yahr (H&Y) IV–V and nonadvanced, H&Y II–III), disease duration (≤ 5, 6–10 and > 10), and use of rehabilitation (used rehab. and nonuse of rehab.). The *X* ^2^ or Fisher’s exact test was used to assess the differences among these selected variables. To focus the results on the more prevalent functioning problems and environmental factors, a threshold of 20% was set. Therefore, entities with at least 20% of patients reporting impairment, difficulty, facilitator, or barrier were included in the results. To avoid Type 1 error (false positive) due to the multiple testing of differences among the selected variables on several functioning problems, the Hochberg correction was performed [26]. The Hochberg correction offers a better balance between Type 1 and Type 2 error risks unlike the Bonferroni correction, which increases the risk of Type 2 error (false negative) [26]. Therefore, the Hochberg sequential procedure was used, and the *p* value was corrected to *p* < 0.001 (i.e., 0.05/34 for impairments; 0.05/62 for activity and participation) [26].

### 2.7. Ethics Approval and Informed Consent Statement

The study received ethics approval from the Stellenbosch University Health Research Ethics Committee (S22/11/256 (PhD)), Ghana Health Service Ethics Review Board (GHS‐ERC: 014/05/23), and the institutional ethics review boards of the teaching hospitals (TH1‐IRB 00087/2023; CCTHERC/EC/2023/152; TH2‐REC (06) FC_2023). Permission was obtained from the heads and management of the included hospitals. Written informed consent was obtained from participants prior to data collection. To avoid confusion between the usual care received at the clinic and the study, no data were collected on the day of recruitment (i.e., participants’ clinic day). In addition, the study was conducted in accordance with the ethical guidelines and principles of the International Declaration of Helsinki and the Medical Research Council (MRC) Ethical Guidelines for Research in South Africa and Ghana. A written informed consent for publication was also obtained from the participants.

## 3. Results

### 3.1. Participant Recruitment and Response Rate

**FIGURE 1:**
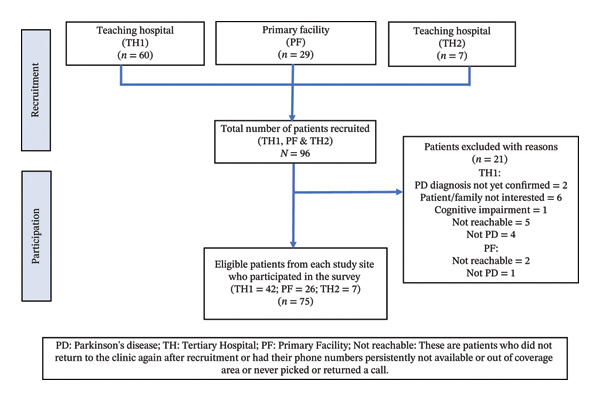
Flow chart for patient recruitment.

Overall, 75 (78.1%) of the 96 recruited PwPD were eligible for inclusion in the study. A varying number of PwPD were included from the different sites, TH1, TH2 and PF, and exclusion was based on the reasons detailed in Figure 1.

### 3.2. Demographic and Brief Health Information of Participants

Table 1 presents the demographic and health information for 75 PwPD enrolled in the study. Of note, there were more males (61.3%), mean (SD) age of 66.8 (9.6) years, and the majority were in H&Y Stage II (62.7%) with a mean (SD) disease duration of 6.0 (4.9) years and mostly retired (58.7%). Approximately half of the participants have not used any form of rehabilitation, and among those who had, physiotherapy was the only rehabilitation used by those participants. The data on age across the three study sites were normally distributed (Shapiro–Wilks test = 0.99; *p* = 0.77), whereas disease duration data were skewed (Shapiro–Wilks test = 0,90; *p* < 0.001). There was no significant difference among the participants from the three study sites based on age (*F* = 0.68, *p* = 0.93), gender (Fisher’s exact test = 2.49, *p* = 0.29), disease duration (*H* = 0.89, *p* = 0.64), and stage (Fisher’s exact test = 5.15, *p* = 0.51). Therefore, further analysis focused on the entire sample rather than the study sites.

**TABLE 1:** Demographic and brief health information details of participants.

Items		Overall *N* = 75	TH1 *n* = 42	PF *n* = 26	TH2 *n* = 7
Gender [*n* (%)]	Male	46 (61.3)	23 (54.8)	17 (65.4)	6 (85.7)
	Female	29 (38.7)	19 (45.2)	9 (34.6)	1 (14.3)

Age (years)	Mean (SD)	66.8 (9.6)	66.7 (8.5)	67.4 (11.9)	66.0 (7.3)
	Min–Max	43–90	52–90	43–89	55–77

MMSE score	Mean (SD)	27.8 (1.8)	28.1 (1.7)	27.1 (1.9)	28.9 (0.7)
	Min–Max	24–30	24–30	24–30	28–30

Level of education [*n* (%)]	No formal education	10 (13.3)	2 (4.8)	8 (30.8)	0
	Junior high school	19 (25.3)	12 (28.6)	7 (26.9)	0
	Senior high school	18 (24.0)	11 (26.2)	5 (19.2)	2 (28.6)
	“O”‐level	6 (8.0)	5 (11.9)	1 (3.8)	0
	Tertiary education	22 (29.3)	12 (28.6)	5 (19.2)	5 (71.4)

Marital status [*n* (%)]	Currently married	40 (53.3)	27 (64.3)	9 (34.6)	4 (57.1)
	No partner	35 (46.7)	15 (35.7)	17 (65.4)	3 (42.9)

Current occupation [*n* (%)]	Employed	13 (17.4)	8 (19.0)	3 (11.5)	2 (28.6)
	Unemployed	18 (24.0)	8 (19.0)	9 (34.6)	1 (14.3)
	Retired	44 (58.7)	26 (61.9)	14 (53.8)	4 (57.1)
PD duration (years)	Mean (SD)	6.0 (4.9)	5.5 (4.5)	6.5 (5.7)	6.9 (5.0)
	Min–Max	0.08–20.00	0.08–19.00	0.08–20.00	1.10–16.00

H&Y stages [*n* (%)]	II	47 (62.7)	28 (37.3)	14 (53.8)	5 (71.4)
	III	16 (21.3)	9 (21.4)	6 (23.1)	1 (14.3)
	IV	10 (13.3)	3 (7.1)	6 (23.1)	1 (14.3)
	V	2 (2.7)	2 (4.8)	0	0

Physical health [*n* (%)]	Very good	3 (4.0)	2 (4.8)	0	1 (14.3)
	Good	23 (30.7)	15 (35.7)	7 (26.9)	1 (14.3)
	Moderate	41 (54.7)	21 (50.0)	15 (57.7)	5 (71.4)
	Bad	8 (10.7)	4 (9.5)	4 (15.4)	0

Mental health [*n* (%)]	Very good	4 (5.3)	1 (2.4)	2 (7.7)	1 (14.3)
	Good	38 (50.7)	27 (64.3)	9 (34.6)	2 (28.6)
	Moderate	30 (40.0)	13 (31.0)	13 (50.0)	4 (57.1)
	Bad	3 (4.0)	1 (2.4)	2 (7.7)	0

Use of rehabilitation [*n* (%)]	No	42 (56.0)	24 (57.1)	15 (57.7)	3 (42.9)
	Yes (PT)	33 (44.0)	18 (42.9)	11 (42.3)	4 (57.1)

*Note:* Min–Max, minimum–maximum.

Abbreviations: H&Y, Hoehn and Yahr; MMSE, Mini‐Mental State Examination; ”O”‐level, ordinary level; PD, Parkinson’s disease; PF, primary facility; PT, physiotherapy; SD, standard deviation; TH, tertiary hospital.

### 3.3. Functioning Problems of Participants Described Using the ICF Classification

Overall, 90 entities out of the 122 were reported as body function (29/33) and structure (5/6) impairments, activity limitations (25/44), participation restrictions (29/44), facilitators (28/39), and barriers (9/39) by at least 20% of the study sample. Entities that did not reach the 20% prevalence threshold can be viewed in Supporting Information 3.

### 3.4. Prevalence of Functioning Problems and Contextual Factors

**FIGURE 2:**
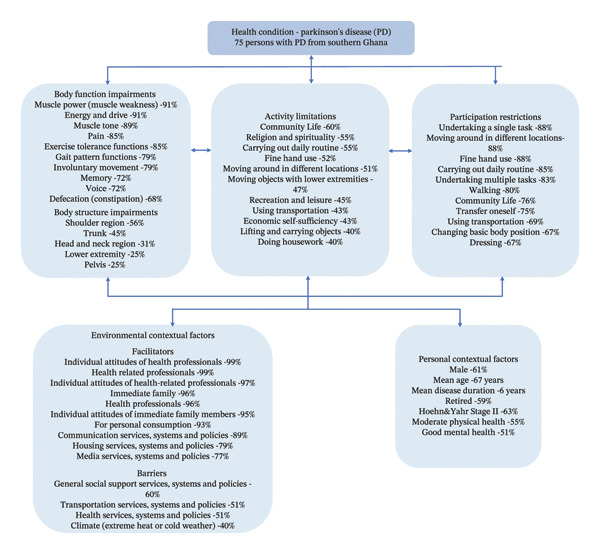
Most prevalent functioning problems and contextual factors in Parkinson’s disease illustrated with the ICF framework.

The most prevalent functioning problems and contextual factors are presented in Figure 2 using the ICF framework. The most prevalent body function impairments were reported by 68%–91% of the participants. Deviating shoulder was the most common body structure impairment reported by 56% of the participants. All the participants reported deviating positions for all body structure impairment entities (six items) except two (2.7%) participants who had abnormal lower extremity shape because of edema. See the figure with all body function and structure impairment variables that met the 20% threshold in Supporting Information 4a.

The most reported challenges were related to participation restrictions. The top 10 participation restriction entities were reported by 67%–88% of the participants, while the top 10 activity limitation entities were reported by 40%–60% of the participants. See the figure with all activity limitation and participation restriction variables that met the 20% prevalence threshold in Supporting Information 4b.

Most of the entities were perceived as facilitators with only nine entities posing as barriers to the participants. The most reported facilitators by over 90% of the participants were mainly within support and relationships (e3), attitudes and services (e4), and systems and policies (e5) chapters. The most reported barrier by 60% of the participants was challenges with general social support. See the figure with all variables (facilitators and barriers) that met the 20% prevalence threshold in Supporting Information 4c.

### 3.5. Substantial Functioning Problems

#### 3.5.1. Body Function and Structure Impairments

**FIGURE 3:**
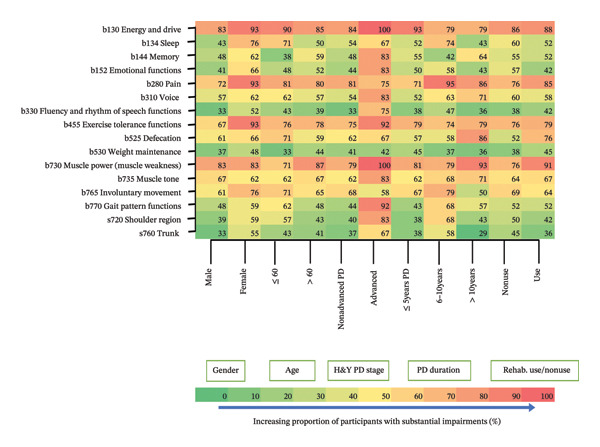
Proportion of participants with substantial body function and structure impairments stratified by selected variables. *b* = body function; *s* = body structure.

The majority of the top 10 most prevalent and substantial body function impairment entities were within the mental function (b1) and neuromusculoskeletal and movement‐related function (b7) chapters. Figure 3 shows the proportion of participants reporting substantial impairments in specific body function and structure entities (reported by ≥ 40% of participants) stratified by gender, age, disease stage, disease duration, and use/nonuse of rehabilitation (full graph in Supporting Information 5). Generally, most participants with advanced PD reported substantial impairments. Energy and drive (79%–100%), pain (71%–95%), voice (52%–83%), exercise tolerance function (67%–92%), defecation (52%–86%), muscle power (71%–100%), muscle tone (62%–83%), and involuntary movement (50%–79%) entities caused substantial impairments to most of the participants with similar proportions across the selected variables (Supporting Information 6a). However, the proportion of participants with advanced versus nonadvanced disease stages differed significantly on joint mobility, involuntary movement reaction, and control of voluntary movement functions at *p* < 0.001 and pelvis and lower extremity structures at *p* < 0.001 (Supporting Information 6a).

#### 3.5.2. Activity Limitations and Participation Restrictions

**FIGURE 4:**
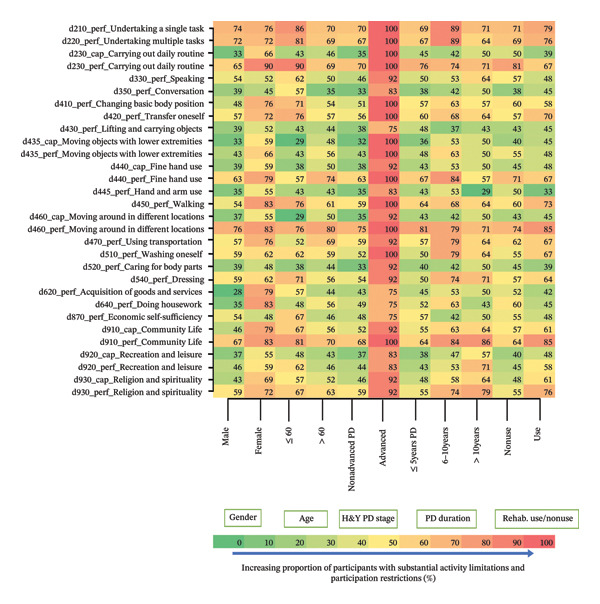
Proportion of participants with significant activity limitations and participation restrictions stratified by selected variables. cap = capacity (activity); perf; performance (participation).

Generally, most of the substantial and prevalent activity limitation (A) and participation restriction (P) entities were within the general task and demand (d2), mobility (d4) and community, and social and civic life (d9) chapters. Figure 4 shows the proportion of participants reporting substantial limitations in activity and restrictions in participation entities (*reported by ≥ 40% of participants*) stratified by gender, age, disease stage, disease duration, and use/nonuse of rehabilitation (full graph in Supporting Information 7). A similar pattern with the body function and structure impairments is observed as most participants with the advanced PD stage reported substantial activity limitations and participation restrictions. Undertaking a single (70%–100%) and multiple (67%–100%) tasks, carrying out daily routines (65%–100%), transfer oneself (56%–100%), fine hand use (57%–100%), walking (54%–100%), moving around in different locations (71%–100%), using transportation (57%–92%), washing oneself (50%–100%), dressing (50%–92%), community life (64%–100%), and religion and spirituality (55%–92%) entities caused substantial participation restrictions to most participants across the selected variables. However, fine hand use (38%–92%), community life (46%–92%), recreation and leisure (37%–83%), and religion and spirituality (43%–92%) entities caused substantial activity limitations to most participants across the selected variables.

A significantly (*p* < 0.001) greater proportion of the female participants had substantial activity limitations and participation restrictions on the acquisition of goods and services, preparation of meals, and doing housework than male participants (Figure 4) (Supporting Information 8). In addition, a greater proportion of participants in the advanced disease stage reported substantial activity limitations (A) and participation restrictions (P) than those in nonadvanced disease stages (Figure 4) and differed significantly (*p* < 0.001) on multiple entities including undertaking single and multiple tasks (A), carrying out daily routines (A), changing basic body positions (A&P), lifting and carrying objects (A), moving objects with lower extremities (A&P), fine hand use (A), walking (A), moving around different locations (A), using transportation (A), washing oneself (A), caring for body parts (P), and dressing (A) (Supporting Information 8). For entities within the 20% threshold, there were no significant differences among participants in varied age groups, disease duration, and use/nonuse of rehabilitation.

## 4. Discussion

The study has described the most prevalent and substantial functioning problems of PwPD in the southern part of Ghana using the ICF and further stratified by gender, age, disease stage and duration, and use/nonuse of rehabilitation. The prevalent contextual factors that influence functioning were also described.

Body function impairments that were commonly reported and posed substantial problems to 68%–91% of study participants were within the mental functioning and neuromusculoskeletal and movement‐related function chapters. These included energy and drive, muscle power and tone, pain, exercise tolerance, gait pattern functions, involuntary movement, memory, voice, and defecation (constipation). Our study found a higher prevalence of impairments (89%–91%) than reported in the previous literature. Raggi et al. [6] reported 78%–79% for involuntary movement and muscle tone impairments among PwPD. Unlike Raggi’s study, which included both in‐ and out‐patients, ours focused solely on out‐patients, who may report more impairments due to greater functional demands. Constipation affected 68% of our participants, lower than the 80% reported by Frazzitta et al. [27]. Per experience, this may be explained by cultural reluctance to report bowel issues unless severe. Constipation, a common nonmotor symptom of PD, often precedes diagnosis and is associated with increased PD risk [28, 29]. This highlights the need for patient education to improve reporting and intervention. Pain, another prevalent nonmotor symptom, was reported by 85% of the participants. However, it may be undertreated since only 18.4% of an Ethiopian study participants received pain medication, and none used physiotherapy [30]. Although our study did not assess pain management, the high prevalence underscores the need for better training in pain assessment and treatment, as physiotherapists may not prioritize it even when PwPD are referred [31]. The most common body structure impairment was protracted (deviating) shoulder. In contrast, Cao and colleagues [32] identified scoliosis, camptocormia, Pisa syndrome, and antecollis as the most prevalent axial deformities. Most of our participants were in H&Y Stage II, which may explain the difference, as those deformities are linked to more advanced stages [32].

Similar proportions of participants, regardless of gender, age, disease duration or stage, and use/nonuse of rehabilitation reported substantial impairments, highlighting the need for comprehensive assessment throughout PD progression. However, those in advanced stages showed more impairments in urinary function, joint mobility, involuntary movement reaction, voluntary movement control, and pelvis and lower extremities. This aligns with the literature from high‐income countries [33, 34], confirming PD’s progressive nature even in underserved regions. These impairments should be prioritized in advanced‐stage assessments.

Most PwPD in this study experienced participation restrictions, reflecting difficulty performing daily tasks [23]. While capacity refers to what one can do when instructed, performance reflects real‐life functioning [35]. Most participants struggled with daily functioning (participation restriction) related to undertaking single and multiple tasks, walking, transferring, and dressing and had limited capacity (activity limitation) for religion and spirituality, moving objects with lower extremities, recreation and leisure, economic self‐sufficiency, and doing housework. Most participants also faced both activity limitations and participation restrictions in mobility, fine hand use, daily routines, community life, and transport. Despite this, common outcome measures (e.g., Berg Balance Scale, 10MWT, and 6MWT) used in PD rehabilitation focus mainly on capacity [8], whereas rehabilitation for conditions such as stroke often targets real‐world performance [36]. Zajac et al. [37] found only a modest link between improved walking capacity and real‐world performance in PwPD. This underscores the need for rehabilitation to address both capacity and performance needs to enhance quality of life.

Most females reported substantial activity limitations and participation restrictions in shopping, meal preparation, and housework, tasks traditionally assigned to women in Ghana [38, 39]. This may explain their greater reported challenges. Therefore, women with PD may benefit from targeted occupational therapy to support their roles as wives, mothers, and grandmothers. Additionally, participants in advanced PD stages reported substantial activity limitations and participation restrictions on multiple entities, reflecting the progressive decline in both motor and nonmotor functions [33, 34]. Amara et al. [40] also observed a steady decline in physical activity over 4 years among early‐stage PwPD. The study findings highlight the need for interventions that promote physical activity to help manage disease symptoms.

The study found that access to personal use products and technology, along with supportive relationships and positive attitudes from family, health and health‐related professionals, was reported as key facilitators for PwPD, helping them navigate daily activities. Given frequent hospital visits, strong healthcare relationships may be vital for treatment adherence [41]. However, participants also faced barriers such as extreme heat, transportation challenges, limited social support, and health issues. Similar studies identified social support from family or friends and professional support as facilitators for exercise, while a lack of exercise partners, poor access, bad weather, and financial constraints were barriers [42, 43]. Anecdotally, Ghana’s public transport poses challenges for PwPD, often forcing patients to hire taxis, which are costly. Although social policies such as retirement income security, elderly healthcare, and livelihood empowerment against poverty exist for the elderly in Ghana, implementation gaps and limited awareness limit their impact [44]. This highlights the need for better policy execution to ease the struggles of PwPD who are mostly elderly. It also underscores the need for clinicians to assess environmental facilitators and barriers unique to each patient for consideration in the care process. Due to disparities in cultures, family systems, and healthcare operational settings, our findings may inform local interventions and policy implementation to reinforce facilitators and overcome barriers.

Using the ICF conceptual framework allowed for the comparison of PwPD functioning in a low‐resource setting with global data. However, the cross‐sectional design, limited geographic scope (southern Ghana), and small sample from TH2 restrict causal inference and generalizability.

In conclusion, impairments in body function and participation restrictions were the most frequently reported functioning problems. Women experienced substantial activity limitations and participation restrictions in home‐related tasks, and those with advanced PD reported greater functioning problems. Facilitators included family and professional support, while barriers involved limited social support and accessibility. Despite some study limitations, the findings offer baseline data to guide comprehensive assessment in PD and highlights critical functioning problems faced by PwPD in resource‐limited settings such as Ghana. It also underscores the value of using the ICF framework in rehabilitation assessment and planning. The findings may inform gender‐ and disease‐stage‐sensitive interventions for PwPD across diverse healthcare contexts.

## Author Contributions

Mary Wetani Agoriwo, Erika Franzén, Marianne Unger, and Conran Joseph contributed equally to the conceptualization of the study. Mary Wetani Agoriwo and Conran Joseph contributed to data curation; Mary Wetani Agoriwo helped in investigation; Mary Wetani Agoriwo, Erika Franzén, Marianne Unger, and Conran Joseph contributed to methodology; Erika Franzén, Marianne Unger, and Conran Joseph supervised the study; Mary Wetani Agoriwo wrote the original draft; and Erika Franzén, Marianne Unger, and Conran Joseph reviewed and edited the manuscript.

## Funding

This work was carried out with the support of the Organization for Women in Science for the Developing World (OWSD); Swedish International Cooperation Agency (Sida) under Fund Reservation No. 3240378593; Transforming Parkinsons Care in Africa (TraPCAf) project under Grant No. NIHR133391; Stellenbosch University Physiotherapy Division Bursary‐PG Strategic Fund under Grant No. PSF‐2022; Mawazo Fellowship Programme under Grant No. 2023‐1‐03; and Harry Crossley Foundation Research Grant under Grant No. HCG‐2024.

## Disclosure

The funders had no influence on the conduct, analysis, and interpretation of study findings. The authors declare that *“An unauthorized version of the MMSE was used by the study team without permission, however this has now been rectified with PAR. The MMSE is a copyrighted instrument and may not be used or reproduced in whole or in part, in any form or language, or by any means without written permission of PAR*.*”*

## Conflicts of Interest

The authors declare no potential conflicts of interest with respect to the research, authorship, and/or publication of this article. The first author received funding from various sources to support her PhD and research, but these funders had no influence on the conduct, analysis and interpretation of study findings.

## Supporting Information

Additional supporting information can be found online in the Supporting Information section.

## Supporting information


**Supporting Information** Supporting Information 1: List of removed and added ICF items. The table provides a list of ICF entities that were excluded (not directly related to PD) and those that were added for the survey. Supporting Information 2: Demographic data of pilot participants. This provides details of the demographic data of the pilot participants. This includes information on gender, age, disease duration, education level, marital status, occupation, and Mini‐Mental State Examination score. Supporting Information 3: Entities that did not meet the 20% prevalence threshold. The table presents details on ICF entities reported by less than 20% of the participants as impairments, activity limitations, participation restrictions, facilitators, or barriers. Supporting Information 4: Variables that met the 20% prevalence threshold. 4a: Body function and structure impairment variables that met the 20% prevalence threshold. The figure shows the body function and structure variables that were reported by ≥ 20% of the participants as impairments. 4b: Activity limitation and participation restriction variables that met the 20% prevalence threshold. The figure shows the activity and participation variables that were reported by ≥ 20% of the participants as limitations or restrictions, respectively. 4c: Facilitator and barrier variables that met the 20% prevalence threshold. The figure shows the variables that were reported by ≥ 20% of the participants as facilitators and barriers. Supporting Information 5: Full graph of proportion of participants with substantial body function and structure impairments stratified by selected variables. This figure provides the full graph of the proportion of participants with substantial body function and structure impairments stratified by gender, age, disease stage and duration, and use/nonuse of rehabilitation. Gender was categorized as male and female, age as ≤ 60 and > 60 years, disease stage as advanced and nonadvanced, disease duration as ≤ 5, 6–10, and > 10 years, and use or nonuse of rehabilitation. Supporting Information 6: Differences in participants’ body function and structure impairment stratified by gender, age, disease stage and duration, and use/nonuse of rehabilitation. The table presents the differences in participants’ body function and structure impairment based on their gender, age, disease stage and duration, and use/nonuse of rehabilitation. Gender was categorized as male and female, age as ≤ 60 and > 60 years, disease stage as advanced and nonadvanced, disease duration as ≤ 5, 6–10, and > 10 years, and use or nonuse of rehabilitation. Supporting Information 7: Full graph of proportion of participants with significant activity limitations and participation restrictions stratified by selected variables. This figure provides the full graph of the proportion of participants with significant activity limitations and participation restrictions stratified by gender, age, disease stage and duration, and use/nonuse of rehabilitation. Gender was categorized as male and female, age as ≤ 60 and > 60 years, disease stage as advanced and nonadvanced, disease duration as ≤ 5, 6–10, and > 10 years, and use or nonuse of rehabilitation. Supporting Information 8: Differences in participants’ activity limitation and participation restriction stratified by gender, age, disease stage and duration, and use/nonuse of rehabilitation. The table presents the differences in participants’ activity limitation and participation restriction based on their gender, age, disease stage and duration, and use/nonuse of rehabilitation. Gender was categorized as male and female, age as ≤ 60 and > 60 years, disease stage as advanced and nonadvanced, disease duration as ≤ 5, 6–10, and > 10 years, and use or nonuse of rehabilitation.

## Data Availability

The data that support the findings of this study are available from the corresponding author upon reasonable request.
