# Supplementary material for: ICF‐Based Assessment of Functioning Problems in Parkinson’s Disease: Findings From a Cross‐Sectional Survey in Southern Ghana
Source: Parkinsons Dis. 2026 May 29;2026:4054078. doi: 10.1155/padi/4054078 (PMC13240471; doi:10.1155/padi/4054078)
Supplement: Supplementary file 1 — Supporting Information Supporting Information 1: List of removed and added ICF items. The table provides a list of ICF entities that were excluded (not directly related to PD) and those that were added for the survey. Supporting Information 2: Demographic data of pilot participants. This provides details of the demographic data of the pilot participants. This includes information on gender, age, disease duration, education level, marital status, occupation, and Mini‐Mental State Examination score. Supporting Information 3: Entities that did not meet the 20% prevalence threshold. The table presents details on ICF entities reported by less than 20% of the participants as impairments, activity limitations, participation restrictions, facilitators, or barriers. Supporting Information 4: Variables that met the 20% prevalence threshold. 4a: Body function and structure impairment variables that met the 20% prevalence threshold. The figure shows the body function and structure variables that were reported by ≥ 20% of the participants as impairments. 4b: Activity limitation and participation restriction variables that met the 20% prevalence threshold. The figure shows the activity and participation variables that were reported by ≥ 20% of the participants as limitations or restrictions, respectively. 4c: Facilitator and barrier variables that met the 20% prevalence threshold. The figure shows the variables that were reported by ≥ 20% of the participants as facilitators and barriers. Supporting Information 5: Full graph of proportion of participants with substantial body function and structure impairments stratified by selected variables. This figure provides the full graph of the proportion of participants with substantial body function and structure impairments stratified by gender, age, disease stage and duration, and use/nonuse of rehabilitation. Gender was categorized as male and female, age as ≤ 60 and > 60 years, disease stage as advanced and nonadvanced, disease durat [file PADI-2026-4054078-s001.docx]

# Supplementary material

## Supplementary material 1: List of removed and added ICF Items

**Justification for removing some items**

The ICF checklist is not disease specific, hence some of the items were considered not to be directly related to Parkinson’s disease (PD) in basic daily functioning were excluded. Additional relevant entities identified from earlier scoping review and the MDS-UPDRS were included.

| **Impairments of Body Functions** | | |
| --- | --- | --- |
| **Removed Items** | ***Added Items*** | |
| **b1. Mental Functions** |  | |
| **b110** Consciousness |  | |
| **b117** Intellectual (incl. Retardation, dementia) |  | |
| **b167** Mental functions of Language |  | |
| **b2. Sensory Functions and Pain** | | |
|  | ***b255** Smell function (sensing odour and smells; anosmia or hyposmia) | |
|  |  | |
| **b3. Voice and Speech Functions** |  | |
|  | ***b320** Articulation functions | |
|  | (Hypokinetic dysarthria - difficulty speaking because of weak speech muscles, Loss of clarity of speech;) | |
|  | ***b330** Fluency and rhythm of speech functions (Rapid speech rate; Stammer - freezing of speech, repetitive speech cadence, monotone speech) | |
| **b4. Functions of the Cardiovascular, Haematological, Immunological and Respiratory Systems** | | |
| **b410** Heart | ***b455** Exercise tolerance functions (physical endurance, aerobic capacity, stamina and fatigue) | |
| **b430** Haematological (blood) |  | |
| **b435** Immunological (allergies, hypersensitivity) |  | |
| **b5. Functions of the Digestive, Metabolic and Endocrine Systems** | | |
| **b515** Digestive | ***b510** Ingestion functions (chewing and biting, manipulating food in the mouth, salivation, swallowing, drooling) | |
| **b555** Endocrine glands (hormonal changes) | ***b535** Sensations associated with the digestive system (sensations of nausea, feeling bloated, abdominal cramps, fullness of stomach,) | |
| **b7. Neuromusculoskeletal and Movement Related Functions** | | |
|  | ***b755** Involuntary movement reaction functions (postural reactions, balance reactions – to prevent falls) | |
|  | ***b760** Control of voluntary movement functions (control and coordination problems; right left motor coordination, eye-hand coordination) | |
|  | ***b770** Gait pattern functions (festinating gait) | |
| **B8. Functions of the Skin and Related Structures** |  | |
|  |  | |
| ***Body Structures*** | | |
| **Removed Items** | | ***Added Items*** |
| **S1. Structure of the Nervous System** | |  |
| **s110** Brain | |  |
| **s120** Spinal cord and peripheral nerves | |  |
| **s2. The Eye, Ear and Related Structures** | |  |
| **S3. Structures Involved in Voice and Speech** | |  |
| **S4. Structure of the Cardiovascular,** | |  |
| **Immunological and Respiratory Systems** | |  |
| **s410** Cardiovascular system | |  |
| **s430** Respiratory system | |  |
| **S5. Structures Related to the Digestive, Metabolism and Endocrine Systems** | |  |
| **S6. Structure Related to Genitourinary and Reproductive System** | |  |
| **s610** Urinary system | |  |
| **s630** Reproductive system | |  |
|  | |  |
| **Activity Limitations & Participation Restriction** | | |
| **Removed Items** | ***Added Items*** | |
| **d1. Learning and Applying Knowledge** |  | |
| d110 Watching |  | |
| d115 Listening |  | |
| d140 Learning to read |  | |
| d145 Learning to write |  | |
| d150 Learning to calculate (arithmetic) |  | |
| d175 Solving problems |  | |
| **d2. General Tasks and Demands** | | |
|  | ***d230** Carrying out daily routine | |
| **d4. Mobility** | | |
|  | ***d410** Changing basic body position (getting into and out of a body position – lying down, sitting, standing, squatting, bending etc.) | |
|  | ***d415** Maintaining a body position (lying down, sitting, standing, squatting, bending) | |
|  | ***d420** Transferring oneself (Moving from one surface to another without changing position - transferring oneself while sitting or lying eg. moving from a wheelchair to a car seat, mobility in bed) | |
|  | ***d435** Moving objects with lower extremities (kicking) | |
|  | ***d445** Hand and arm use (throwing, catching) | |
|  | ***d460** Moving around in different locations | |
| **d6. Domestic Life** |  | |
| d660 Assisting others |  | |
| **d7. Interpersonal Interactions and Relationships** |  | |
| d710 Basic interpersonal interactions |  | |
| d720 Complex interpersonal interactions |  | |
| d730 Relating with strangers |  | |
| **d8. Major Life Areas** | | |
| d810 Informal education | ***d845** Acquiring, keeping and terminating a job | |
| d820 School education |  | |
| **d9. Community, Social and Civic Life** |  | |
| d940 Human rights |  | |
| d950 Political life and citizenship |  | |
|  |  | |
| **Section 2b: Contextual Factors**  **Environmental Factors** | | |
| **Removed Items** | ***Added Items*** | |
| **e1. Products and Technology** | | |
|  | ***e130** Products and technology for education (general and assistive products and technology for education) | |
|  | ***e165** Assets (own property and other valuables, financial assets, etc.) | |
| **e3. Support and Relationships** | | |
|  | ***e315** Extended family (aunts, uncles, nephews and nieces) | |
| **e4. Attitudes** | | |
|  | ***e415** Individual attitudes of extended family members | |
|  | ***e425** Individual attitudes of acquaintances, peers,  colleagues, neighbours and community members | |
|  | ***e430** Individual attitudes of people in positions of authority | |
| **e5. Services, Systems and Policies** | | |
|  | ***e560** Media services, systems and policies (mass communication through radio, television, newspapers and internet) | |

## Supplementary material 2: Demographic Data of pilot participants

| Items | | Overall  N=8 | TH 1  N=4 | TH 2,  N=4 |
| --- | --- | --- | --- | --- |
| Gender | Male | 4 | 2 | 2 |
|  | Female | 4 | 2 | 2 |
| Age | Mean (SD) | 68.4 (6.0) | 68.5 (6.5) | 68.3 (5.4) |
| PD duration (years) | Median (range) | 2.25 (0.25-4.0) | 3 (0.25-3) | 1.25 (1-4) |
| Hoehn & Yahr stage | I | 1 | 1 | 0 |
|  | II | 6 | 2 | 4 |
|  | III | 1 | 1 | 0 |
| Level of education | No formal education | 2 | 2 | 0 |
|  | Junior high school | 2 | 1 | 1 |
|  | Senior high school | 1 | 0 | 1 |
|  | Tertiary | 3 | 1 | 2 |
| Marital status | Never married | 1 | 0 | 1 |
|  | Currently Married | 2 | 1 | 1 |
|  | Divorced | 1 |  | 1 |
|  | Widowed | 4 | 3 | 1 |
| Current occupation | Non-paid work, such as volunteer/charity | 1 | 1 | 0 |
|  | Retired | 7 | 3 | 4 |
| MMSE Score | Mean (SD) | 27.3 (2.0) | 26.3 (2.2) | 28.3 (1.3) |

PD=Parkinson’s disease; SD=Standard deviation; MMSE=Mini Mental State Examination; TH=Teaching Hospital

## Supplementary material 3: Categories that did not meet the 20% prevalence threshold

| **Items** | **Number of participants n=75** |
| --- | --- |
| **Body function categories** |  |
| b114_ Orientation (time, place, person)* | 11 |
| b210_ Seeing (ability to see shape, color, size, etc., vision problems) | 11 |
| b156_ Perceptual functions (Hallucination or illusion, psychosis) | 7 |
| b230_ Hearing (deafness, hearing impairment and hearing loss) | 1 |
| **Body structure categories** |  |
| s730_ Upper extremity *(arm, hand)* | 6 |
| **Activity and participation** |  |
| d350_cap_Conversation | 13 |
| d845_perf_Acquiring, keeping and terminating a job | 12 |
| d315_perf_Communicating with -- receiving -- non-verbal messages | 11 |
| d335_perf_ Producing non-verbal messages | 11 |
| d415_cap_ Maintaining a body position (lying down, sitting, standing, squatting, bending) | 11 |
| d465_perf_ Moving around using equipment *(moving in a wheelchair or with walker, etc.)* | 11 |
| d530_perf_Toileting (able to use and cleaning oneself afterwards) | 11 |
| d550_perf_Eating (able to eat served food, bring to mouth, cutting and breaking into pieces) | 11 |
| d570_cap_Looking after one`s health | 11 |
| d845_cap_Acquiring, keeping and terminating a job | 11 |
| d850_perf_Remunerative employment | 11 |
| d335_cap_Producing non-verbal messages | 10 |
| d530_cap_Toileting (able to use and cleaning oneself afterwards) | 10 |
| d850_cap_Remunerative employment | 10 |
| d310_cap_Communicating with -- receiving -- spoken messages | 8 |
| d315_cap_Communicating with -- receiving -- non-verbal messages | 8 |
| d550_cap_Eating (able to eat served food, bring to mouth, cutting and breaking into pieces) | 8 |
| d560_perf_Drinking | 8 |
| d740_perf_Formal relationships (eg. relations with work colleagues, employees or employers, professionals or service providers) | 8 |
| d770_perf_Intimate relationships (romantic, spousal and sexual relationships) | 8 |
| d855_perf_ Non-remunerative employment | 8 |
| d855_cap_ Non-remunerative employment | 8 |
| d465_cap_Moving around using equipment *(moving in a wheelchair or with walker, etc.)* | 7 |
| d770_cap_Intimate relationships (romantic, spousal and sexual relationships) | 7 |
| d560_cap_Drinking | 6 |
| d740_cap_ Formal relationships (eg. relations with work colleagues, employees or employers, professionals or service providers) | 6 |
| d760_perf_Family relationships (parent-child and child-parent relationships, sibling and extended family relationships) | 6 |
| d750_perf_Informal social relationships (eg. relations with friends, neighbours, acquaintances, co-inhabitants) | 4 |
| d760_cap_Family relationships (parent-child and child-parent relationships, sibling and extended family relationships) | 4 |
| d830_perf_Higher education (Bachelor’s, Master’s, PhDs) | 4 |
| d750_cap_Informal social relationships (eg. relations with friends, neighbours, acquaintances, co-inhabitants) | 2 |
| d830_cap_Higher education (Bachelor’s, Master’s, PhDs) | 2 |
| **Contextual factors (Environmental factors)** |  |
| *e315_b_Extended family (aunts, uncles, nephews and nieces.) | 13 |
| *e415_b_Individual attitudes of extended family members | 11 |
| e150_f_Design, construction and building products and technology of buildings for public use (access) | 10 |
| e155_b_Design, construction and building products and technology of buildings for private use (access) | 10 |
| e525_b_Housing services, systems and policies | 9 |
| e460_f_Societal attitudes | 8 |
| e570_b_Social security, services, systems and policies | 8 |
| e465_f_Social norms, practices and ideologies | 6 |
| e575_f_General social support services, systems and policies (policies on support for shopping, housework, transport, self-care and care of others) | 6 |
| e590_f_Labour and employment services, systems and policies | 6 |
| e590_b_Labour and employment services, systems and policies | 5 |
| *e130_f_Products and technology for education (general and assistive products and technology for education) | 4 |
| e250_f_Sound (noisy or quiet environment) | 4 |
| *e425_b_Individual attitudes of acquaintances, peers, colleagues, neighbours and community members | 4 |
| e585_f_Education and training services, systems and policies | 4 |
| e125_b_Products for communication (voice amplifiers) | 3 |
| e125_f_Products for communication (voice amplifiers) | 3 |
| e240_b_Light (lighting in the environment especially at night) | 3 |
| e310_b_Immediate family (spouses, partners, parents, siblings, children, foster parents, etc.) | 3 |
| e320_b_Friends | 3 |
| e325_b_Acquaintances, peers, colleagues, neighbors and community members | 3 |
| e330_b_People in position of authority | 3 |
| e340_b_Personal care providers and personal assistants | 3 |
| e410_b_Individual attitudes of immediate family members | 3 |
| e420_b_Individual attitudes of friends | 3 |
| e535_b_Communication services, systems and policies | 3 |
| *e560_b_Media services, systems and policies (mass communication through radio, television, newspapers and internet) | 3 |
| e110_b_For personal consumption (food and medicines availability) | 2 |
| e225_f_Climate (extreme heat or cold weather) | 2 |
| e440_b_Individual attitudes of personal care providers and personal assistants | 2 |
| e120_b_For personal indoor and outdoor mobility and transportation (eg. canes, walkers, wheelchair) | 1 |
| *e130_b_Products and technology for education (general and assistive products and technology for education) | 1 |
| e355_b_Health professionals (eg. nurses, doctors, physiotherapists, etc.) | 1 |
| *e430_b_Individual attitudes of people in positions of authority | 1 |
| e550_f_Legal services, systems and policies | 1 |
| e115_b_For personal use in daily living (availability of assistive devices) | 0 |
| e360_b_Health related professionals (eg, records staff, etc) | 0 |
| e450_b_Individual attitudes of health professionals | 0 |
| e455_b_Individual attitudes of health-related professionals | 0 |
| e550_b_Legal services, systems and policies | 0 |
| e585_b_Education and training services, systems and policies | 0 |

## Supplementary material 4: Variables that met the 20% prevalence threshold

### 4a: Body function and structure impairment variables that met the 20% prevalence threshold


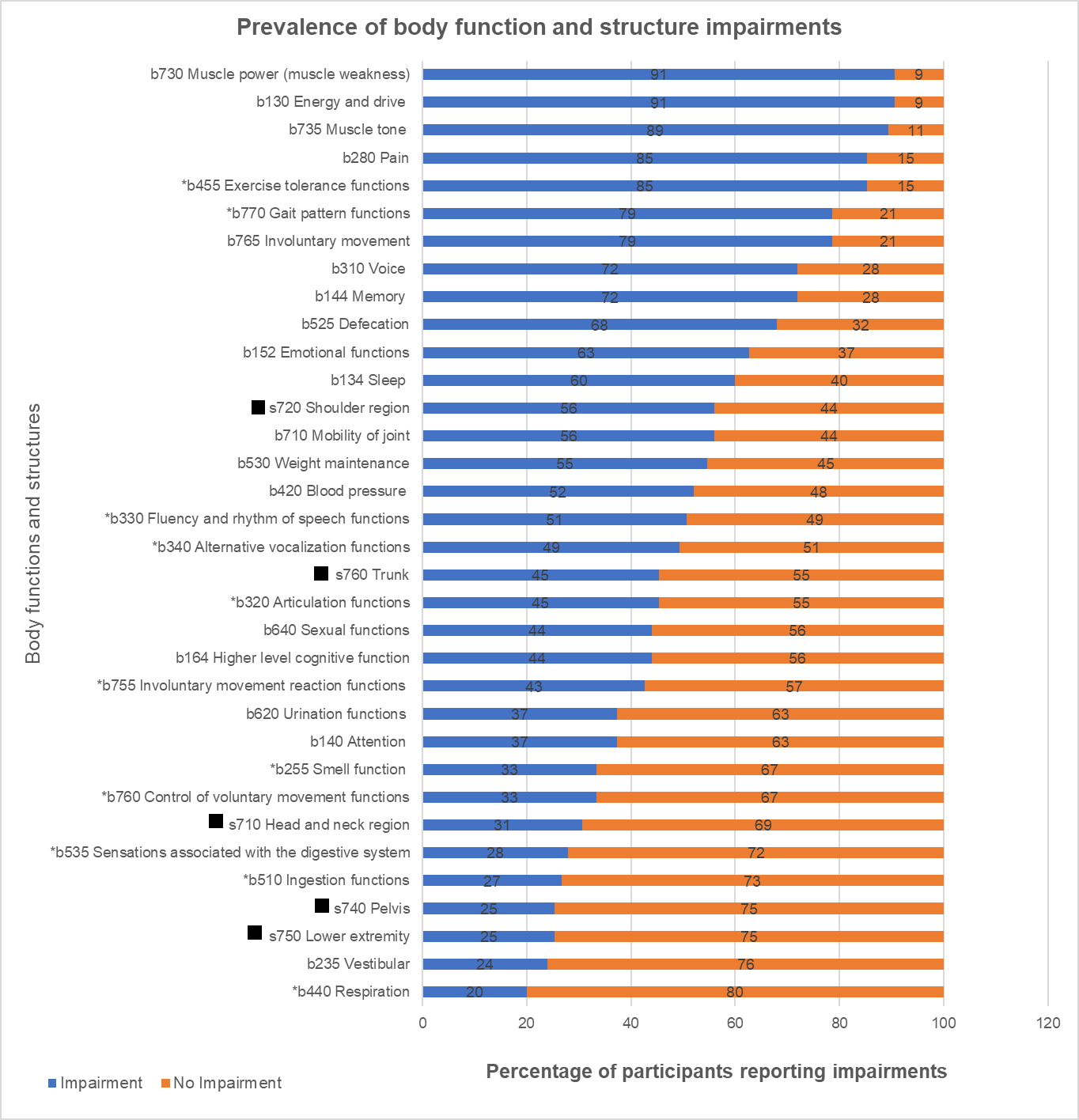


Body structure

### 4b: Activity limitation and participation restriction variables that met the 20% prevalence threshold


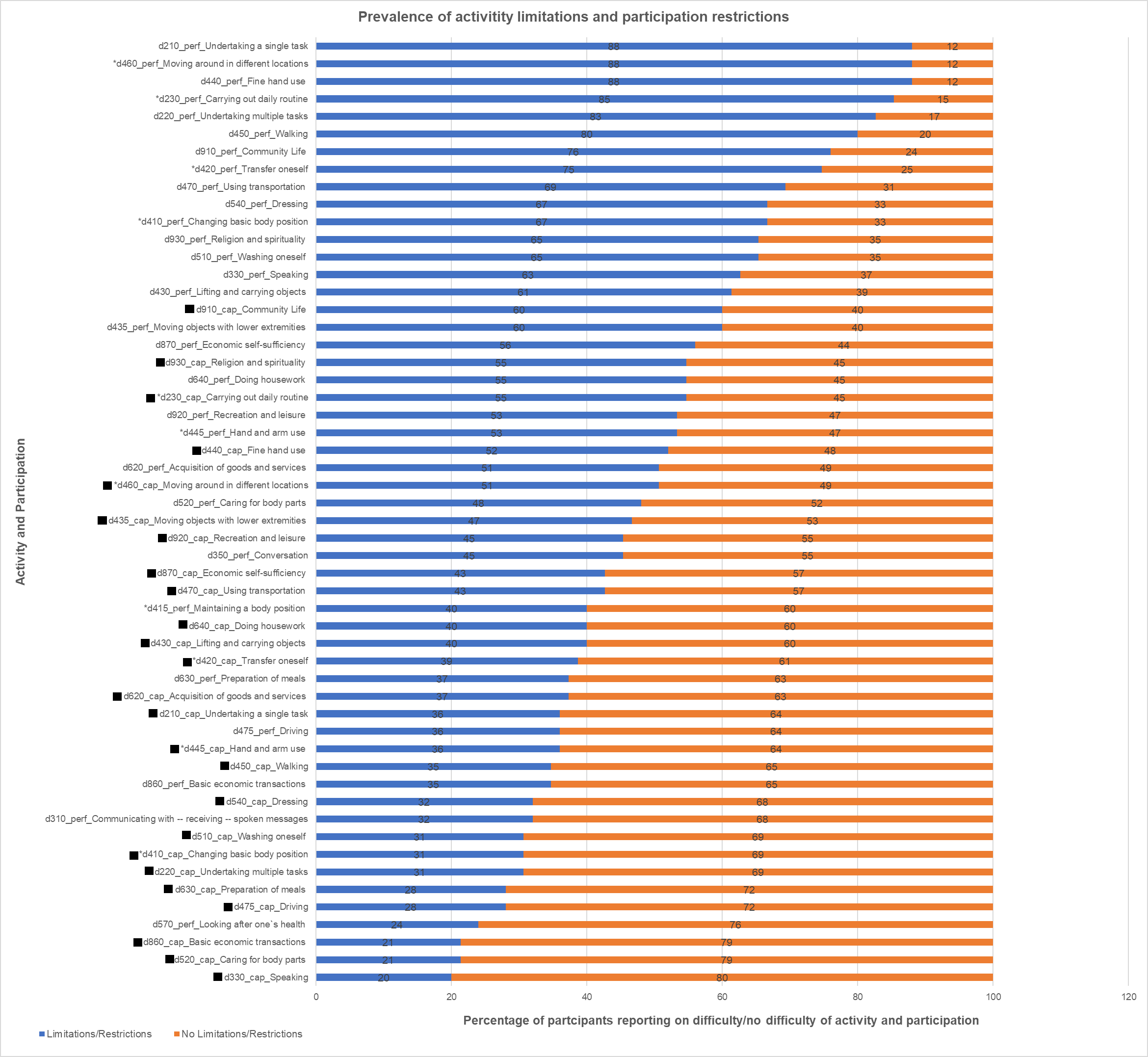


Capacity (Activity limitation)

### 4c: Facilitators and barriers variables that met the 20% prevalence threshold


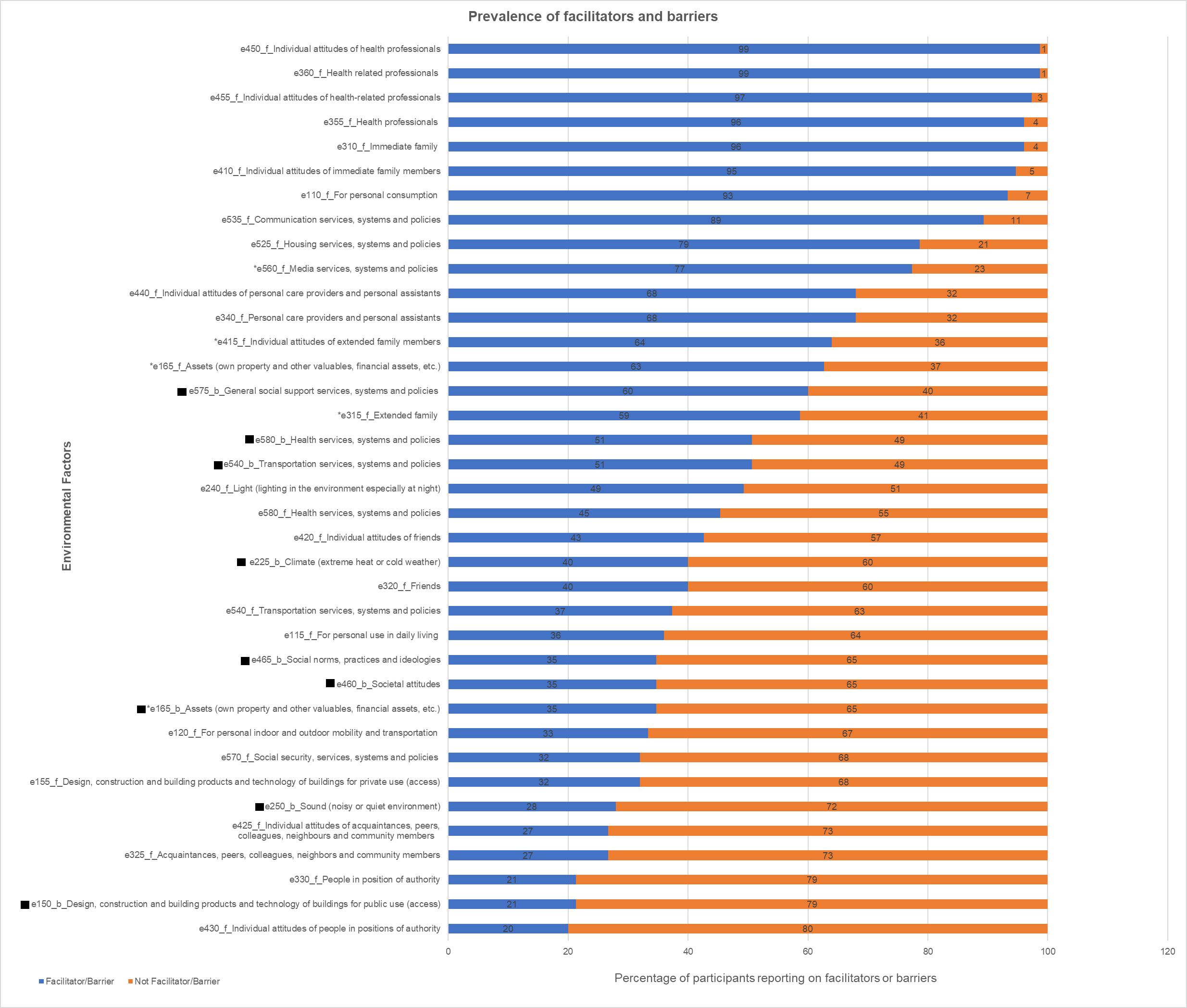


Barrier

## Supplementary material 5: Full graph of proportion of participants with substantial body function and structure impairments stratified by selected variables

b=body function; s=body structure

## Supplementary material 6: Differences in participants’ body function and structure impairment stratified by gender, age, disease stage and duration and use/non-use of rehabilitation

## Supplementary material 7: Full graph of proportion of participants with significant activity limitations and participation restrictions stratified by selected variables

cap= capacity (activity); perf; performance (participation)

## Supplementary material 8: Differences in participants’ activity limitation and participation restriction stratified by gender, age, disease stage and duration and use/non-use of rehabilitation

## Checklist for Reporting of Survey Studies (CROSS)

| **Section/topic** | **Item** | **Item description** | **Reported on page #** |
| --- | --- | --- | --- |
| **Title and abstract** | | |  |
| Title and abstract | 1a | State the word “survey” along with a commonly used term in title or abstract to introduce the study’s design. | 1 |
|  | 1b | Provide an informative summary in the abstract, covering background, objectives, methods, findings/results, interpretation/discussion, and conclusions. | 2 |
| **Introduction** | | |  |
| Background | 2 | Provide a background about the rationale of study, what has been previously done, and why this survey is needed. | 3-4 |
| Purpose/aim | 3 | Identify specific purposes, aims, goals, or objectives of the study. | 4 |
| **Methods** | | |  |
| Study design | 4 | Specify the study design in the methods section with a commonly used term (e.g., cross-sectional or longitudinal). | 4-5 |
|  | 5a | Describe the questionnaire (e.g., number of sections, number of questions, number and names of instruments used). | 5-6 |
| Data collection methods | 5b | Describe all questionnaire instruments that were used in the survey to measure particular concepts. Report target population, reported validity and reliability information, scoring/classification procedure, and reference links (if any). | 5-6 |
|  | 5c | Provide information on pretesting of the questionnaire, if performed (in the article or in an online supplement). Report the method of pretesting, number of times questionnaire was pre-tested, number and demographics of participants used for pretesting, and the level of similarity of demographics between pre-testing participants and sample population. | 6 |
|  | 5d | Questionnaire if possible, should be fully provided (in the article, or as appendices or as an online supplement). | To be made available upon request |
| Sample characteristics | 6a | Describe the study population (i.e., background, locations, eligibility criteria for participant inclusion in survey, exclusion criteria). | 5 |
|  | 6b | Describe the sampling techniques used (e.g., single stage or multistage sampling, simple random sampling, stratified sampling, cluster sampling, convenience sampling). Specify the locations of sample participants whenever clustered sampling was applied. | 5 |
|  | 6c | Provide information on sample size, along with details of sample size calculation. | 5 |
|  | 6d | Describe how representative the sample is of the study population (or target population if possible), particularly for population-based surveys. | 5 |
| Survey  administration | 7a | Provide information on modes of questionnaire administration, including the type and number of contacts, the location where the survey was conducted (e.g., outpatient room or by use of online tools, such as SurveyMonkey). | 6-7 |
|  | 7b | Provide information of survey’s time frame, such as periods of recruitment, exposure, and follow-up days. | 5 |
|  | 7c | Provide information on the entry process:  –>For non-web-based surveys, provide approaches to minimize human error in data entry.  –>For web-based surveys, provide approaches to prevent “multiple participation” of participants. | 6-7 |
| Study preparation | 8 | Describe any preparation process before conducting the survey (e.g., interviewers’ training process, advertising the survey). | 6 |
| Ethical considerations | 9a | Provide information on ethical approval for the survey if obtained, including informed consent, institutional review board [IRB] approval, Helsinki declaration, and good clinical practice [GCP] declaration (as appropriate). | 8 |
|  | 9b | Provide information about survey anonymity and confidentiality and describe what mechanisms were used to protect unauthorized access. | 7 |
| Statistical  analysis | 10a | Describe statistical methods and analytical approach. Report the statistical software that was used for data analysis. | 7 |
|  | 10b | Report any modification of variables used in the analysis, along with reference (if available). |  |
|  | 10c | Report details about how missing data was handled. Include rate of missing items, missing data mechanism (i.e., missing completely at random [MCAR], missing at random [MAR] or missing not at random [MNAR]) and methods used to deal with missing data (e.g., multiple imputation). | No missing data |
|  | 10d | State how non-response error was addressed. | N/A |
|  | 10e | For longitudinal surveys, state how loss to follow-up was addressed. | N/A |
|  | 10f | Indicate whether any methods such as weighting of items or propensity scores have been used to adjust for non-representativeness of the sample. | N/A |
|  | 10g | Describe any sensitivity analysis conducted. | N/A |
| **Results** | | |  |
| Respondent characteristics | 11a | Report numbers of individuals at each stage of the study. Consider using a flow diagram, if possible. | 9 |
|  | 11b | Provide reasons for non-participation at each stage, if possible. | 9 |
|  | 11c | Report response rate, present the definition of response rate or the formula used to calculate response rate. | 8 |
|  | 11d | Provide information to define how unique visitors are determined. Report number of unique visitors along with relevant proportions (e.g., view proportion, participation proportion, completion proportion). | N/A |
| Descriptive  results | 12 | Provide characteristics of study participants, as well as information on potential confounders and assessed outcomes. | 8-16 |
| Main findings | 13a | Give unadjusted estimates and, if applicable, confounder-adjusted estimates along with 95% confidence intervals and p-values. | N/A |
|  | 13b | For multivariable analysis, provide information on the model building process, model fit statistics, and model assumptions (as appropriate). | 7 |
|  | 13c | Provide details about any sensitivity analysis performed. If there are considerable amount of missing data, report sensitivity analyses comparing the results of complete cases with that of the imputed dataset (if possible). | N/A |
| **Discussion** | | |  |
| Limitations | 14 | Discuss the limitations of the study, considering sources of potential biases and imprecisions, such as non-representativeness of sample, study design, and important uncontrolled confounders. | 19 |
| Interpretations | 15 | Give a cautious overall interpretation of results, based on potential biases and imprecisions and suggest areas for future research. | 19 |
| Generalizability | 16 | Discuss the external validity of the results. | 19 |
| **Other sections** | | |  |
| Role of funding source | 17 | State whether any funding organization has had any roles in the survey’s design, implementation, and analysis. | 20 |
| Conflict of interest | 18 | Declare any potential conflict of interest. | 19 |
| Acknowledgements | 19 | Provide names of organizations/persons that are acknowledged along with their contribution to the research. | 20 |
